# Toxoplasmosis Is More Frequent in Schizophrenia Patients Than in the General Population in Mexico and Is Not Associated with More Severe Course of Schizophrenia Measured with the Brief Psychiatric Rating Scale

**DOI:** 10.3390/pathogens10070820

**Published:** 2021-06-30

**Authors:** María de la Luz Galván-Ramírez, Gabriela Navarro Machuca, Sergio Armando Covarrubias Castillo, Juan Carlos Benavides González, Laura Roció Rodríguez Pérez, Sergio Horacio Dueñas Jiménez, Judith Marcela Dueñas Jiménez

**Affiliations:** 1 Departamento de Microbiología y Patología, Centro Universitario de Ciencias de la Salud, Universidad de Guadalajara, Guadalajara C.P. 45100, JA, Mexico; jucabengo@hotmail.com (J.C.B.G.); laura.rperez@academicos.udg.mx (L.R.R.P.); sduenas@cucs.udg.mx (S.H.D.J.); mduenas@cucs.udg.mx (J.M.D.J.); 2 Servicio de Psiquiatría del Hospital Civil Fray Antonio Alcalde, Guadalajara C.P. 44280, JA, Mexico; gabynava@hotmail.com (G.N.M.); scovarrubias@hcg.gob.mx (S.A.C.C.)

**Keywords:** *Toxoplasma gondii*, *Toxoplasma* infection, schizophrenic patients, prevalence, anti-*Toxoplasma* antibodies

## Abstract

Toxoplasmosis is a disease, which was discovered in 1908, caused by the intracellular parasite *Toxoplasma gondii*. *T. gondii* infects neuronal, glial, and muscle cells, and chronic infections are characterized by the presence of cysts, in the brain and muscle cells, formed by bradyzoites. *T. gondii* is capable of synthesizing L-DOPA, a precursor of dopamine. Dopamine is a neurotransmitter that is key in the etiology of neuropsychological disorders such as schizophrenia. Previous studies have shown high levels of IgG *Toxoplasma* antibodies in schizophrenia patients. Many published studies show that the prevalence of toxoplasmosis is higher in schizophrenia patients. In this study, we aimed to identify the prevalence of *Toxoplasma* infection in patients with schizophrenia and the relationships between, sociodemographic factors and the Brief Psychiatric Rating Scale. A total of 27 schizophrenic patients were included and IgG anti-*T. gondii* was determined in serum samples by ELISA. The Brief Psychiatric Rating Scale, sociodemographic factors were associated with seropositivity. We found that the prevalence of *Toxoplasma* antibodies was 51.7%. In the Brief Psychiatric Rating Scale, statistical significant association (*p* = 0.024) was found in Item 13 which is related to motor retardation, however, the association turned non-significant after of correction for multiple tests or after of analyzed with a logistic regression *p* = 0.059, odds ratio (OR) = 2.316 with a 95% confidence interval [0.970 to 5.532]. Other association was not found between toxoplasmosis and others factors. The prevalence of toxoplasmosis on our population under study was significantly higher than that reported by general population or other group of Mexican schizophrenia patients.

## 1. Introduction

Toxoplasmosis is a disease, which was discovered in 1908, caused by an intracellular protozoan called *Toxoplasma gondii*. The infection can be acquired by various mechanisms such as vertical transmission from mother to child and orally through cysts present in raw or undercooked meat [1] and oocysts present in water or fruit and vegetables watered with sewage water and eaten without hand washing. Other infecting mechanisms are infected organ transplants, blood transfusions, and direct contamination, when working in laboratories with hand wounds or when contaminated raw meat is handled [2]. Recently, a new route of infection has been suggested that toxoplasmosis could also be transmitted sexually and by oral sex [3]. 

The genome of *T. gondii* contains two genes encoding tyrosine hydroxylase that produces levodopa (L-DOPA), which is a precursor to dopamine. The encoded enzymes metabolize phenylalanine as well as tyrosine with a preference for the tyrosine substrate. One of the genes of *Toxoplasma*, TgAaaH1, is constitutively expressed, while the other gene, TgAaaH2, is induced by the bradyzoite formation during the life cycle of cyst formation, inducing high levels of dopamine in brain infected with *Toxoplasma* which can produce some schizophrenic symptoms [4]. 

Other studies have detected that *T. gondii* produced L-DOPA in brain of infected rodents [5]. This has led to the hypothesis that an increase in dopamine during infection is associated with observed behavioral changes [5,6,7,8]. Treatment with a dopamine inhibitor (GBR 12909), has been shown to alter the behavior of *T. gondii*-infected mice [9].

Schizophrenia is a multifactorial disease, which can cause a disabling neuropsychological condition; there is a high incidence of schizophrenia in the general population. Many studies published over the last 20 years show strong association between latent toxoplasmosis and Schizophrenia [10,11]. The first study conducted in schizophrenia patients with primary episodes found that anti-*Toxoplasma* antibodies were significantly higher as compared with that in a control group [12]. 

With respect to epidemiology, a prospective follow-up study by Butajira, in Addis Ababa, Ethiopia, of 80 patients with schizophrenia, showed that 87.9% of the patients were positive for anti-*T. gondii* antibodies class IgG [13]. In France, a study of patients with schizophrenia found that 184 (73.6%) of the patients had latent *Toxoplasma* infection associated with an increased risk in specific symptoms, for example, delusion OR = 3.6 (1.2–10.6) (*p* = 0.01) [14]. In Iran, 99 patients with schizophrenia and 41 patients with a suicide attempt were studied and IgG anti-*Toxoplasma* antibodies were positive in 42% of the patients with schizophrenia and 27% of the patients with a suicide attempt, with a significant difference of *p* = 0.04. [15]. In a Russian study of 155 patients with schizophrenia and 152 healthy people in a control group, IgM and IgG anti-*Toxoplasma* were determined. In both groups, IgM was negative but IgG anti-*Toxoplasma* antibodies were positive in 40% of patients and 25% of the control group. The absence of IgM immunoglobulin and the presence of IgG suggested a latent form of toxoplasmosis in both the patient and control groups [16]. In a Brazil study, IgG and IgM antibodies determined that there was a higher prevalence of anti-*T. gondii* IgG in patients with schizophrenia than in a control group (91.18% vs. 70.59% of the control group) (*p* = 0.017). Interestingly, in this study, IgM anti-*T. gondii* antibodies the marker of an acute form of toxoplasmosis were not detected in any patient studied [17]. A cross-sectional study was conducted, in Egypt, with 177 individuals, where IgG and IgM anti-*T. gondii* antibodies from schizophrenic patients and a control group were determined. *Toxoplasma* antibodies among schizophrenic patients were higher (31.75%) as compared with the controls (14.55%). Only three patients, all with schizophrenia, had positive IgM antibodies [18]. 

A study conducted in Mexico, involving 137 hospitalized patients diagnosed with schizophrenia, 93 patients with acute psychiatric illness and 44 patients with chronic psychiatric disease, found that 25/137 (18.2%) of the psychiatric patients and 16/180 of the controls (8.9%) were positive for IgG anti-*T. gondii* antibodies. There was no statistically significant difference between acute and chronic schizophrenia [19]. Another study, also carried out in Mexico, used meta-analysis and found that the average prevalence of anti-*Toxoplasma* antibodies was 27.97% and the weighted prevalence was 19.27%; however, it was almost two times higher in mentally ill patients (38.52%) [20]. 

The Brief Psychiatric Rating Scale (BPRS), developed by Overall and Gorham (1962), is used to assess changes in the symptoms of psychiatric patients. Depending on the version of the scale, there are a total of 18–24 symptoms and each symptom is rated on a scale from one to seven points [21].

In this study, we analyzed the prevalence of *Toxoplasma* infection in patients with schizophrenia and searched for the association of the *Toxoplasma* seropositivity with sociodemographic factors and the traits monitored by the Brief Psychiatric Rating Scale (BPRS).

## 2. Results

### 2.1. Anti-Toxoplasma Antibodies

Among 27 patients, the ELISA test results for *Toxoplasma* antibodies were negative in 12 and positive in 15 patients’ samples, which corresponded to a prevalence of 51.7%; Was higher than 27.97% in the general population, X^2^ = 32.42, (*p* < 0.0001); Is was also higher that prevalence reported in mentally ill patients 38.52%, X^2^ = 10.76, *(p* < 0.0005).

### 2.2. Brief Psychiatric Rating Scale (BPRS)

No association was detected between having high BPRS score (20 or more points on the BPRS scale) and being *Toxoplasma* seropositive and seronegative.

The relative frequencies (percentages) of the responses given by the patients, 15 positive and nine negative to *Toxoplasma*, as well as the items on the BPRS scale were analyzed using the Mann–Whitney test, no association significant differences in any symptom item of the BPRS with Fisher’s exact test (*p* = 0.057). When the mean scores for the BPRS items and the positivity for anti-*Toxoplasma* antibodies were analyzed using the Mann–Whitney test for the total of 27 patients, 18 items for 24 patients were analyzed and we found a statistically significant difference in Item 13 related to motor retardation with (*p* = 0.024). However, the association turned non-significant after of correction for multiple tests or after of analyzed with a logistic regression multivariate *p* = 0.059, odds ratio (OR) = 2.316 with a 95% confidence interval 2.316 [0.970 to 5.532] Table 1). 

### 2.3. Sociodemographic Data

There was a total of 12 women and 15 men in the sample studied. The distribution by age group was similar, i.e., approximately one-third of the patients were younger than 30, one-third were in the range from 30 to 49 years old, and the remaining third of adults were aged 50 and over. Non-statistically significant differences were observed in the distribution of age groups between genders with the chi-square test (X^2^) (*p* = 0.869) or with Fisher’s exact test (*p* = 0.893). The mean age of the women was 46.3 years with a standard deviation of ±17.5 years, the minimum age was 20 years, and the maximum age was 71 years; in the case of the men, the mean age was 38.3 ± 14.6 years with a minimum age of 16 years and a maximum age of 64 years. The median ages were 47.5 years for women and 33 years for men. When comparing the ages of men and women to the Student’s t-test and the Mann–Whitney non-parametric test, we did not observe any statistically significant differences (*p* = 0.207 and *p* = 0.261, respectively).

Concerning the schooling of the patients studied, 10 patients had elementary school studies, 5 patients had middle school studies, 4 patients had high school studies, only 1 patient had a college degree, and there were 7 patients who did not answer the question concerning schooling. Regarding occupation, the majority of patients did not work (22 cases), four patients were employees, and one patient was a merchant. 

The associations between anti-*Toxoplasma* antibodies and sociodemographic characteristics (age, gender) were analyzed using contingency tables and no statistically significant effect was detected Table 2.

**Figure 1:**
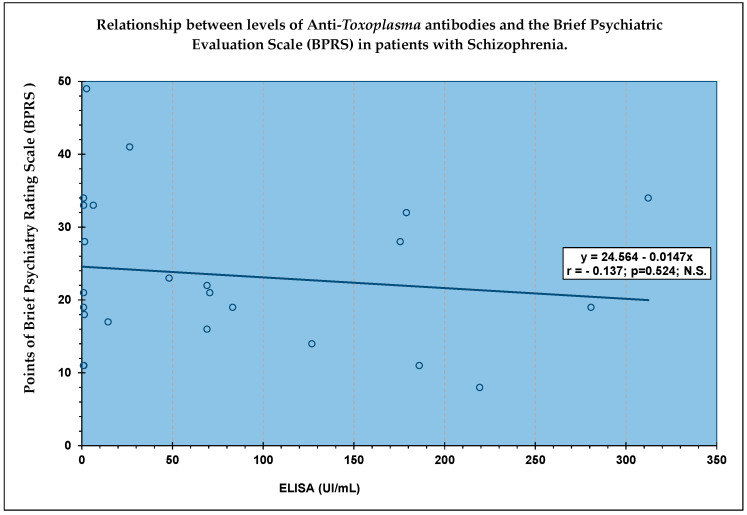
No significant correlation between the ELISA test results and the Brief 160 Evaluation Scale Psychiatric (BPRS) score in 24 patients.

Finally, when using the Pearson and Spearman correlation coefficient between the ELISA results (UI/mL) and the Brief Psychiatric Assessment Scale to assess 24 schizophrenia patients, there was no relationship between these two measurements Figure 1.

## 3. Discussion

In our study, we found the current prevalence was 51.7%, which was higher than 27.97% in the general population, X^2^ = 32.42, (*p* < 0.0001); Is was also higher that prevalence reported in mentally ill patients the prevalence was 38.52%, which was significantly higher, X^2^ = 10.76, *(p* < 0.0005) [20,22].

We only determined IgG antibodies, since most studies have shown that *Toxoplasma* seropositivity in patients with schizophrenia was mainly associated with chronic infection [13,16,19]. There have been studies where IgM and IgG antibodies were determined, however, no patient was positive for IgM [17] or a minimal number of such subjects was present [18].

In this study, we investigated the associations of toxoplasmosis with each item on the BPRS. No association was significant after the correction for multiple tests. *Toxoplasma* infection of the brain undoubtedly affects neuro-inflammatory processes, including microglial activation, the levels of inflammatory cytokines, and the number of peripheral immune cells occurring during the infection. All these biochemical and cellular processes alter behavior in various ways and might affect the risk of schizophrenia and the course of the disease [23,24,25].

Our study, indeed, found an increased prevalence of toxoplasmosis in schizophrenia patients, however, the number of participants was probably not high enough for the detection of the effect of toxoplasmosis on the course of the disease.

Elevated antibody levels have also been linked to schizophrenia as compared with other patient groups [23,24]. The results of our study did not show a statistically significant correlation with antibody levels. One explanation for our results may be that they are latent-stage infections with low levels of IgG antibodies and a reactivation did not occur in which IgGs rise exponentially. Another possibility may be the different methods reported and their cut-off values [23], the probable explanation of this difference is the very small number of patients involved in our study.

## 4. Materials and Methods

### 4.1. Patients

The inclusion criterion was the provision of a blood sample, which was obtained from 29 patients diagnosed with schizophrenia at the Psychiatric Service of the “Fray Antonio Alcalde” Civil Hospital in Guadalajara, Jalisco, Mexico, from March to August 2019. That met the criteria of the *Statistical Diagnostic Manual*, fourth edition [21].

The exclusion criteria excluded patients who had a history of drug use or whose family did not accept their participation.

### 4.2. Clinical Aspects

The data obtained from patients were immunodeficiency, evidence of neurological disease, blood transfusion, transplants, behavior, and treatment.

### 4.3. Brief Psychiatric Rating Scale (BPRS)

The items on the Brief Psychiatric Rating Scale include the following; BPRS01, somatic concern; BPRS02, psychic anxiety; BPRS03, emotional isolation; BPRS04, conceptual disorganization (incoherence); BPRS05, self-contempt and guilt feelings; BPRS06, tension, somatic anxiety; BPRS07, mannerism and strange Postures; BPRS08, greatness; BPRS09, depressive humor; BPRS10, hostility; BPRS11, suspicion; BPRS12, hallucinations; BPRS13, motor retardation; BPRS14, lack of cooperation; BPRS15, unusual thought content; BPRS16, dullness, affective flattening; BPRS17, excitement; BPRS18, disorientation, confusion. Scores and meaning of the responses to the items comprising the Brief Psychiatric Assessment Scale (BPRS) are as follows: (1) not present, (2) very mild, (3) mild, (4) moderate (5) moderate-severe, (6) serious, and (7) very serious.

### 4.4. Sociodemographic Factors

The sociodemographic factors included age, birthplace, residence, marital status, occupation, educational level, and socioeconomic level [26].

### 4.5. Serological Testing for T. gondii Antibodies

Blood samples were processed in the Neurophysiology Laboratory at the University Center for Health Sciences, University of Guadalajara. The serum samples were obtained by centrifugation and kept frozen at –20 °C until they were processed. IgG anti-*T. gondii* (Platelia TM Toxo, Bio-Rad, Marnes-la-Coquette, France) antibody titers were determined in all samples by ELISA. Plates were read at 450/620 nm. Optical density values obtained were plotted along a standard curve to determine the levels of antibody titers (IU/mL). Samples with titers less than 6 IU/mL were considered to be negative and those with titers ranging from 9 to 200 IU/mL were considered to be positive for latent infection. Samples with values greater than 200 IU/mL were checked twice. All tests were performed in accordance with the manufacturer’s instructions.

### 4.6. Statistical Analysis

SPSS Version 20.0 and SPSS (v. 18) packages (IBM, Los Angeles, CA, USA) were used to perform all statistical analysis. Quantitative variables included age, the BPRS scores, and the ELISA test results. The Shapiro–Wilk test of “normality” was applied as well as measures of central and dispersion tendency for the seropositivity ELISA results. The statistical significance between the two groups (positive vs. negative to ELISA) for the differences observed in these variables was obtained with the Student’s t-test for independent samples and with the Mann–Whitney U test. We calculated the Pearson (r) and Spearman (Rho) correlation coefficient between the ELISA values and the BPRS test scores. Linear regression was also performed between the total BPRS score and the ELISA values of these subjects.

The qualitative variables included age groups (under 30 years, 30–49 years, and 50 and older). There were two BPRS groups, i.e., schizophrenic patients with a BPRS score greater than or equal to 20 points and those patients with a score of less than 20 points.

Qualitative variables were analyzed using contingency tables, contrasting positive and negative results for ELISA from patients. The statistical significance with Fisher’s exact test and the odds ratio were also calculated. Multivariate analysis, namely the logistic regression, was also used for the assessment of the others factors.

## 5. Conclusions

In conclusion, the prevalence of latent toxoplasmosis observed in schizophrenia patients was significantly higher than that reported earlier for General population in Mexico. We did not found significant effects of toxoplasmosis on the symptoms of schizophrenia measured with BPRS, or a significant association of the *Toxoplasma* infection with any monitored factors. These negative outputs are most probably caused by rather low sample size.

## Figures and Tables

**Table 1:** Mean of items in the Brief Psychiatric Rating Scale (BPRS) and the seropositivity of anti-*Toxoplasma* antibodies.

Items	Mean	±	EE	Mean	±	EE	*p*
Brief Psychiatric Rating Scale (BPRS)	Positive (>6 UI/mL)			Negative (<6 UI/mL)			Mann–Whitney
BPRS01, somatic concern	2.20	±	0.38	2.11	±	0.56	0.808
BPRS02, psychic anxiety	2.67	±	0.41	3.78	±	0.80	0.286
BPRS03, emotional Isolation	3.00	±	0.47	2.67	±	0.60	0.694
BPRS04, conceptual disorganization (inconsistency)	3.20	±	0.52	3.89	±	0.68	0.439
BPRS05, self-contempt and feelings of guilt	1.87	±	0.50	1.78	±	0.40	0.828
BPRS06, tension and somatic anxiety	2.67	±	0.45	3.11	±	0.61	0.549
BPRS07, mannerism and strange postures	2.20	±	0.55	2.11	±	0.56	0.885
BPRS08, greatness	1.87	±	0.50	1.78	±	0.57	0.903
BPRS09, depressive humor	2.20	±	0.48	2.67	±	0.44	0.310
BPRS10, hostility	2.64	±	0.46	2.78	±	0.70	0.986
BPRS11, suspicion	2.93	±	0.51	3.22	±	0.83	0.961
BPRS12, hallucinations	2.40	±	0.56	3.33	±	0.83	0.33
BPRS13, motor retardation	2.67	±	0.30	1.56	±	0.38	0.024*
BPRS14, lack of cooperation	2.73	±	0.41	2.33	±	0.60	0.412
BPRS15, unusual content of thought	3.53	±	0.54	3.89	±	0.75	0.725
BPRS16, dullness and affective flattening	3.40	±	0.40	3.11	±	0.48	0.687
BPRS17, excitement	2.20	±	0.55	1.56	±	0.29	0.780
BPRS18, disorientation and confusion	1.40	±	0.19	3.22	±	0.83	0.067

**Table 2:** Age, gender and seropositivity of *Toxoplasma gondii by ELISA*, in psychiatric patients.

	No. (+)	%	No. (−)	%	Total	*p*	OR	95% CI
					Cat.			
**Age group**						0.691		
<30 years	4	50	4	50	8	0.391	0.429	[0.062–2.972]
30–49 years	4	44	5	55.6	9	0.266	0.343	[0.052–2.261]
50 and over	7	70	3	30	10	1	1	- - - - -
Total	15	55.6	12	44.4	27			
**Gender**						0.707		
Male	9	60	6	40	15	0.707	1.5	[0.324–6.942]
Female	6	50	6	50	12	1	1	-----------------
Total	15	55.6	12	44.4	27			

## Data Availability

All data is in the manuscript.
